# A Single Mutation at Position 120 in the Envelope Protein Attenuates Tembusu Virus in Ducks

**DOI:** 10.3390/v14030447

**Published:** 2022-02-22

**Authors:** Dawei Yan, Binbin Wang, Ying Shi, Xintao Ni, Xiaogang Wu, Xuesong Li, Xingpo Liu, Haiwang Wang, Xin Su, Qiaoyang Teng, Jianmei Yang, Qinfang Liu, Zejun Li

**Affiliations:** 1 Department of Avian Infectious Diseases, Shanghai Veterinary Research Institute, Chinese Academy of Agricultural Sciences, Shanghai 200241, China; yandawei865@163.com (D.Y.); bbwang321@163.com (B.W.); shiyingsunny@126.com (Y.S.); nxt320482@foxmail.com (X.N.); jason_wu5230@163.com (X.W.); lixuesong@shvri.ac.cn (X.L.); liuxingpo1362@163.com (X.L.); wanghaiwang2022@163.com (H.W.); suxin900220@163.com (X.S.); tengqy@shvri.ac.cn (Q.T.); yangjianmei@shvri.ac.cn (J.Y.); 2 Institute of Animal Husbandry and Veterinary Sciences, Shanghai Academy of Agricultural Sciences, Shanghai 201106, China

**Keywords:** Tembusu virus, envelope protein, attenuation, tissue tropism, transmissibility

## Abstract

A live attenuated duck Tembusu virus (TMUV) vaccine FX2010-180P (180P) was successfully utilized to prevent TMUV infections in ducks in China. Compared with wild-type TMUV, 180P was highly attenuated and lost transmissibility in ducks. However, the mechanism of the attenuation of 180P remains poorly understood. To explore the key molecular basis of attenuation, chimeric and site mutant viruses in the background of the wild-type TMUV-FX2010 (FX) strain were rescued, and the replication, tissue tropism, and transmissibility were characterized in ducks. The results show that the envelope (E) protein was responsible for attenuation and loss of transmission in ducks. Further studies showed that a D120N amino acid mutation located in domain II of the E protein was responsible for the attenuation and transmissibility loss of 180P in ducks. The D120N substitution resulted in an extra high-mannose type N-linked glycosylation (NLG) in the E protein of 180P compared with the wild-type TMUV, which might restrict the tissue tropism and transmissibility of TMUV in ducks. Our findings elucidate that N120 in the E protein is a key molecular basis of TMUV attenuation in ducks and provide new insight into the role of NLG in TMUV tissue tropism and transmissibility.

## 1. Introduction

TMUV belongs to the Ntaya virus group within the genus *Flavivirus*, family *Flaviviridae* [1]. TMUV was first isolated in mosquitoes in Malaysia in 1955 [2]. In 2010, TMUV caused outbreaks in both young and laying ducks, characterized in China for the first time by a retarded growth, severe drop in egg production, and neurologic disorders [3]. TMUV has quickly spread in ducks through the country and has become endemic in China since 2010. Recently, TMUV was detected in Southeast Asia and caused huge economic loss to the poultry industry [4,5,6,7].

Most of the viruses in the genus *Flavivirus*, family *Flaviviridae,* are arboviruses, transmitted by mosquitoes or other arthropods, such as Dengue virus (DENV), Zika virus (ZIKV), Japanese encephalitis virus (JEV), West Nile virus (WNV), etc. However, our previous study showed that the newly emerged TMUV obtained direct-contact and aerosol transmissibility in ducks, which made the virus spread quickly among ducks even in the winter season in Northern China [8]. As we know, TMUV is the first flavivirus in the family Flaviviridae that has aerosol transmissibility among the host, while the underlying mechanism remains unclear. 

To control the TMUV infection in ducks, we had developed a live attenuated TMUV vaccine 180P, licensed in China in 2018. The attenuated 180P vaccine was developed by serially passaging the wild-type FX in chicken embryo fibroblast (CEF) [9]. Compared with the wild-type TMUV, the 180P vaccine was significantly attenuated in ducks and lost the direct-contact transmissibility in ducks. Amino acid alignment results showed there were 19 amino acid mutations in 180P compared with those of FX. However, the underlying molecular mechanisms of the attenuation of 180P remained unclear. 

TMUV E protein plays an important role in tissue tropism and pathogenicity of flaviviruses. The E protein contains three structural domains: β-barrel shaped domain I (DI), which contains residues 1–50, 133–197, and 279–300 and acts as a bridge between domain II (DII) and domain III (DIII); finger-like DII is formed by residues 51–132 and 197–278; Ig-like DIII contains the putative receptor-binding site and has an important role in receptor binding and cell entry [10,11,12,13]. 

Using reverse genetic technology, chimeric or mutant TMUV viruses were generated by switching different domains of E protein between FX and 180P, as described previously [14,15]. The virulence and transmissibility of the chimeric viruses were evaluated in ducks. The results show that a residue mutation D to N at position 120 of the E protein was responsible for the attenuation of the 180P vaccine strain. Further studies show that the D120N mutation introduced an extra high-mannose type NLG, which is a protein modification system and characterized by a high structural diversity of N-linked glycans found among different species [16] in the E protein of 180P. Overall, our study sheds light on the molecular virulence determinants of TMUV, which may provide a new approach to the development of flavivirus vaccines.

## 2. Materials and Methods

### 2.1. Cells and Viruses

Baby Hamster Syrian Kidney (BHK-21) cells were obtained from the American Type Culture Collection (ATCC) and maintained in Dulbecco’s Modified Eagle Medium (DMEM) (Hyclone, Logan, UT, USA), supplemented with 5% fetal bovine serum (FBS) (Biowest, South America origin, Riverside, MO, USA), 100 U/mL of penicillin, and 100 μg/mL of streptomycin (Invitrogen, Carlsbad, CA, USA) at 37 °C in a 5% CO_2_ humidified incubator. Primary duck embryo fibroblast (DEF) cells were generated by using 11-day-old specific pathogen-free (SPF) duck embryonated eggs (bought from Harbin Veterinary Research Institute, Chinese Academy of Agricultural Sciences) and cultured in DMEM (Hyclone, Logan, UT, USA), supplemented with 10% FBS, 100 U/mL of penicillin, and 100 μg/mL of streptomycin at 37 °C in 5% CO_2_. FX (GenBank: MH414568.1) was isolated from diseased ducks and purified three times in specific pathogen-free (SPF) chicken embryonated eggs by limiting dilution method. The 180P was attenuated from serial 180 passages of the FX in CEF. All of the rescued viruses were propagated once on BHK-21 cells, aliquoted, and stored at −80 °C.

### 2.2. Plasmid Construction

To generate the templates for PCR-based virus rescue, four plasmids—pFXT7-1-976, pFXE957-2459, pFX2433-3831, and pFX3656-10991—containing the overlapping fragments of FX were generated and were used as templates for full-length genome amplification. As for 180P, accordingly, the four plasmids p180PT7-1-976, p180PE957-2459, p180P2433-3831, and p180P3656-10991 were constructed, respectively. Additionally, plasmids containing different domain substitutions or single-site mutation in the E protein were constructed using a two-step fusion PCR strategy or site-directed mutagenesis kits (TransGene, Inc., Beijing, China) based on pFXE957-2459 and p180PE957-2459.

### 2.3. PCR-Based Rescue of Mutant Tembusu Viruses

The E gene-switched virus was rescued, as described previously [14]. Briefly, pFXT7-1-976, pFXE957-2459, pFX2433-3831, and pFX3656-10991 were used as templates to amplify four overlapping fragments. In addition, the full-length cDNA with T7 promoter was produced by fusion-PCR, using the overlapping PCR fragments as templates. Then, the cDNAs were transcribed in vitro by using mMESSAGEmMACHINE^®^ T7 Kit (Invitrogen, Carlsbad, CA, USA) to generate the infectious viral RNAs. The RNAs were then purified by lithium chloride precipitation and transfected into 70–80% confluent BHK-21 cells, cultured in 35 mm dishes, at amount of 5 μg using Lipofectamine LTX& Plus Reagent (Invitrogen, Carlsbad, CA, USA). The cell culture medium was changed to DMEM containing 2% FBS at 6 h post-transfection. Virus released into the supernatant was collected when 70–80% transfected cells showed apparent cytopathic effects (CPEs) and was stored at −80 °C. For domain or single-site mutant viruses rescue, p180PE957-2459 was substituted according to modified plasmids. The rescued viruses were amplified on BHK-21 cells for only once, based on the report which showed that the BHK-21 cells were able to be support replication of Tembusu virus [17]. Nine overlapping bases of the PCR products, containing the whole genome, were purified and cloned into pSIMPLE19 vector for sequencing to confirm no unexpected mutation.

### 2.4. Growth Kinetics of TMUVs on DEF Cells

To determine the viral replication of rescued viruses in vitro, primary DEF cells were prepared from 11-day-old SPF duck embryos and cultured in T-25 flask. The cells were infected with mutant TMUVs at a multiplicity of infection (MOI) of 0.001. The infected cells were incubated with DMEM containing 2 % FBS at 37 °C. The supernatant was collected every 12 h post-infection and titrated on BHK-21 cells.

### 2.5. Virus Titration

To determine the virus titer, duck tissue samples were weighed and homogenized in sterile PBS to yield 1:1 (mL/g) homogenates. Tissue homogenates or cell culture samples were clarified by centrifugation at 12,000× *g*, 4 °C for 10 min, and undiluted and 10-fold serially diluted supernatants were titrated onto 70–80% confluent BHK-21 cells in 96-well plates at 37 °C for 2 h. After adsorption, cells were washed and cultured with DMEM with 2% FBS in a humidified chamber with 5% CO_2_ at 37 °C. The lower limit of virus detection was 0.5 log10 TCID50 per 0.1 g tissue. The virus titer was calculated by the method of Reed and Muench.

### 2.6. Duck Experiments

To test the virulence and transmissibility of parental FX, 180P, or the mutant viruses in ducks, groups of 9- to 16-week-old female Shelducks (a local outbred strain) were inoculated intramuscularly (i.m.) with 10^3.5^ TCID_50_ of each virus at volume of 0.2 mL, respectively. As for contact groups, naïve ducks were introduced and co-housed with inoculated ducks one day later. All inoculated and contact ducks were monitored daily for clinical changes. Each experiment was conducted as follows: 

To identify whether the E protein was related to tissue tropism and transmissibility of TMUVs, E gene substitution chimeric virus and parental virus were inoculated i.m. into 6 ducks respectively, and 3 naïve ducks were introduced one day later. In each group, three inoculated ducks were euthanized at 3 dpi by CO_2_ inhalation, and the tissue samples of spleen, lung, kidney, and ovary were collected for viral titration. The sera samples were collected from the remaining inoculated ducks at 0, 2, 4, and 8 dpi and from the contact ducks at 0, 3, 5, 7, and 14 dpc for antibody detection.

To determine the critical domain of E protein impacting the tissue tropism and transmissibility of FX in ducks, the single E domain substituted recombinant viruses (FX/180PE-DI, FX/180PE-DII, and FX/180PE-DIII) were rescued and tested in ducks, as designed above.

To determine the key amino acids attributed to the replication, tissue tropism, and transmission of FX in ducks, viruses with single amino acid substitution located at E-DII were rescued and tested in ducks, as designed above. 

### 2.7. Blocking ELISA

To test the duck sera samples for TMUV antibody, a blocking ELISA method was used, as described previously [18]. Briefly, 96-well ELISA plates were coated with purified TMUV FX and blocked with PBS containing 5% skim milk. Serum samples were 10-fold diluted, added into the wells, and incubated for 1 h at 37 °C. The plates were washed with PBS containing 0.05% Tween-20 (PBST) for 3 times, then mAb 1F5 (1:20) was added into the wells, and the plates were incubated for 1 h at 37 °C. Then the plates with PBST were washed for 3 times, followed by the addition of HRP-conjugated goat anti-mouse IgG (1:2000, Sigma, St. Louis, MO, USA). After incubating for 1 h at room temperature (RT) and washing with PBST for 3 times, 3,3’,5,5’-tetramethyl benzidine was added, and the plates were incubated at RT for 5 min. The reaction was then stopped by adding 0.1 N sulfuric acid. The optical density (OD_450nm_) was measured, and the percentage inhibition (PI) value was calculated: PI (%) = [1 − (OD450 nm of test serum/OD450 nm of negative control serum)] × 100%. When the PI value ≥ 18.4%, the sample was considered as positive. 

### 2.8. Western Blot Assay

To analyze whether the D120N mutation introduced an extra glycosylation in E protein, Western blot assays were performed. Briefly, samples were denatured in 5× sample loading buffer (Beyotime, Shanghai, China) at 99 °C for 10 min. The lysates were then centrifuged for 10 min at 12,000× *g* at 4 °C to remove cell debris. Proteins were separated under denaturing conditions in 10% SDS-polyacrylamide gel electrophoresis (SDS-PAGE) and wet-transferred to nitrocellulose membrane (PALL, East Hills, NY, USA). The membrane was blocked with PBST, containing 5% (*w*/*v*) skim milk, for 1 h at RT. TMUV mAb 2C7 (1:500) was added and incubated at 4 °C overnight. After washing with PBST for 3 times, HRP-linked goat anti-mouse IgG (1:2000, Sigma, St. Louis, MO, USA) was added and incubated for 2 h at RT. The membrane was imaged by using SuperSignal West Femto maximum sensitivity substrate (Thermo Fisher, Waltham, MA, USA).

### 2.9. Glycosylation Pattern Identification 

To identify the Glycans type attached on N120 and N154, BHK-21 cells were plated in 6-well plates (1.0 × 10^6^ cells/well), infected with each indicated virus at a MOI of 0.01, and incubated at 37 °C. At 48 h post-infection, supernatants were collected and the cells were washed with PBS for 3 times and solubilized in RIPA lysis buffer (Beyotime, Shanghai, China) with 1 mM of protease inhibitors PMSF (Beyotime, Shanghai, China) for 3 min. Cell lysates were centrifuged at 12,000× *g*, 4 °C for 10 min, to clear cellular debris. The supernatants and cell lysates aliquots were then treated with Endo H_f_ or PNGase F (NEB, Ipswich, MA, USA) according to the manufacturer’s instructions or without enzyme treatment as a control. Samples were then analyzed under denaturing conditions in 10% SDS-PAGE and Western blotting.

### 2.10. Homology Modeling

To analyze the E protein structure, the images of the E proteins were created with the program Phyre2 [19]. The E protein structure was referred to JEV E protein (PDB accession code 5WSN) and visualized and analyzed with PyMOL.

### 2.11. Statistical Analysis

Viral titers were analyzed using variance (ANOVA) analysis in GraphPad Prism 7.0 version software (GraphPad Software Inc., La Jolla, CA, USA), and a *p* value ≤ 0.05 was considered significant.

## 3. Results

### 3.1. Introduction of 180P E Protein Abolished Transmissibility of Wild-Type TMUV

**Figure 1:**
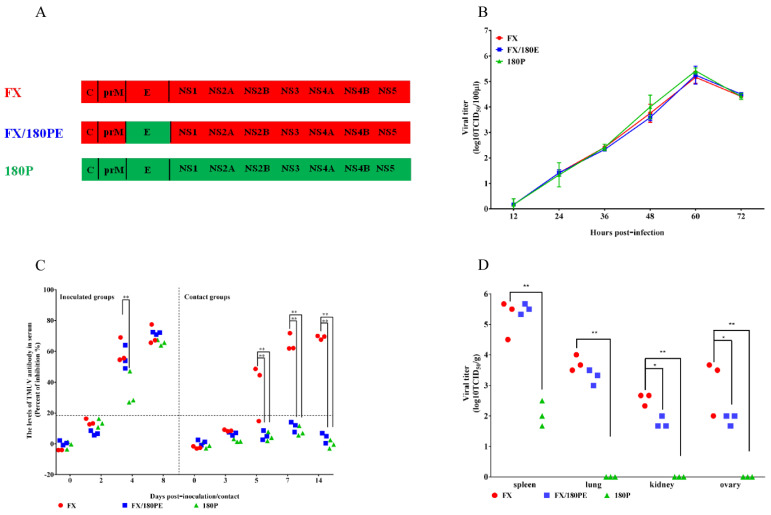
Virus generation, replication, tissue tropism, and transmissibility of parental and E-switched chimeric TMUVs in vitro and in ducks. (**A**) Schematic diagram of chimeric viruses. The colored bars indicate the origin of the viral protein: red, FX; green, 180P. The viruses were rescued in the background of FX. (**B**) Replication of parental and chimeric TMUVs. DEF cells were infected by the parental and E gene substitution TMUVs at a MOI of 0.001. Virus samples from the supernatant were collected at different time points and titrated on BHK-21 cells. The data for virus titers indicate the means of triplicates. (**C**) Antibodies against TMUV in FX-, FX/180PE-, or 180P-inoculated and contact ducks. Serum was considered positive when the PI value was ≥18.4%. (**D**) Virus titers in different samples of ducks inoculated with FX, FX/180PE, or 180P. The data for virus titers indicate the results of 3 ducks. (* *p* < 0.05; ** *p* < 0.01).

There were 19 amino acid mutations located in the entire viral polyprotein of 180P aligned with parental FX, 8 of which were in the E protein (Table 1). To identify whether the E protein was critical to the attenuation and transmissibility of TMUV in vitro and in ducks, the chimeric virus FX/180PE with switched 180P E gene in the background of FX was rescued (Figure 1A) and evaluated on the DEF cells and in ducks. FX/180PE had similar replication capacities as FX on DEF cells infected with each virus at a MOI of 0.001 (Figure 1B). The contact–transmission study showed that all the inoculated ducks seroconverted at 4 days post-inoculation (dpi) (Figure 1C). Seroconversion was only found in contact ducks of the FX group at tested dates. None of the contact ducks in the FX/180PE or 180P groups showed seroconversion even at 14 days post-contact (dpc) (Figure 1C), which indicated that FX/180PE and 180P was not transmitted to the contact ducks. These results indicate that the E protein played a key role in abolishing the transmissibility of 180P. Virus distribution data showed that the chimeric virus FX/180PE and its parental virus FX were detected in the tested spleens, lungs, kidneys, and ovaries of inoculated ducks at 3 dpi, while the virus titers in ovary and kidney of FX/180PE group were lower than those of FX groups (Figure 1D). The results indicate that the E protein contributed to viral replication of TMUV in ducks. In contrast, the 180P vaccine virus was only detected in spleens of inoculated ducks (Figure 1D).

### 3.2. Domain II of E Protein Was Responsible for Both Tissue Tropism and Transmissibility of TMUV in Ducks

**Figure 2:**
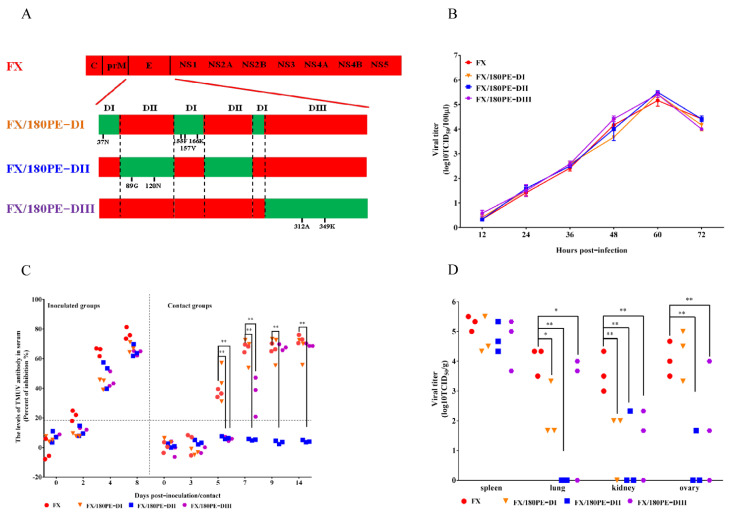
Virus generation, replication, tissue tropism, and transmissibility of parental and E domain-switched chimeric TMUVs in vitro and in ducks. (**A**) Schematic diagram of chimeric viruses. The colored bars indicate the origin of the viral protein: red, FX; green, 180P. The viruses were rescued in the background of FX. (**B**) Replication of parental and E domain-switched TMUVs. DEF cells were infected by the parental and chimeric TMUVs at a MOI of 0.001. Virus samples from the supernatant were collected at different time points and titrated on BHK-21 cells. The data for virus titers indicate the means of the results of triplicates. (**C**) Antibodies against TMUV in FX-, FX/180PE-DI-, FX/180PE-DII-, or FX/180PE-DIII-inoculated and contact ducks. Serum was considered positive when the PI value was ≥18.4%. (**D**) Virus titers in different organs of ducks inoculated with FX, FX/180PE-DI, FX/180PE-DII, or FX/180PE-DIII. The data for virus titers indicate the results of 3 ducks. (* *p* < 0.05; ** *p* < 0.01).

To identify which domain of E protein determined tissue tropism and transmissibility of FX in ducks, the single domain chimeric viruses FX/180PE-DI, FX/180PE-DII, and FX/180PE-DIII that contained DI, DII, and DIII of 180P E protein in the FX backbone were rescued (Figure 2A) and further characterized on DEF cells and in ducks. The chimeric viruses replicated to similar viral titers on DEF cells were compared with wild-type FX (Figure 2B). All ducks inoculated with either one of three chimeric viruses showed seropositive at 4 dpi (Figure 2C). Substitution of single E-DI or E-DIII did not impact transmissibility of FX since the contact ducks from these two groups seroconverted at 7 dpc (Figure 2C), while FX/180PE-DII lost transmissibility in ducks, as none of the contact ducks seroconverted even at 14 dpc (Figure 2C). These data indicate that the DII of the E protein is responsible for transmissibility loss of 180P in ducks. Furthermore, the DII of the E protein was proved to be critical for the virus replication in ducks. All the chimeric viruses replicated to the similar virus titers in duck spleens (Figure 2D). The titers of FX/180PE-DIII were significantly lower in lung, kidney, and ovary compared with that of FX, which suggested that the DIII was important to the replication of the TMUV. However, the chimeric virus containing the E-DII of 180P was undetected in lungs of any inoculated duck, and lower virus loads were detected in kidney and ovary in one out of three infected ducks, which suggested that the FX/180PE-DII virus was largely limited to spleens compared with other viruses (Figure 2D).

### 3.3. The E_D120N_ Mutation Changed the Tissue Tropism and Abolished Transmissibility of TMUV in Ducks

**Figure 3:**
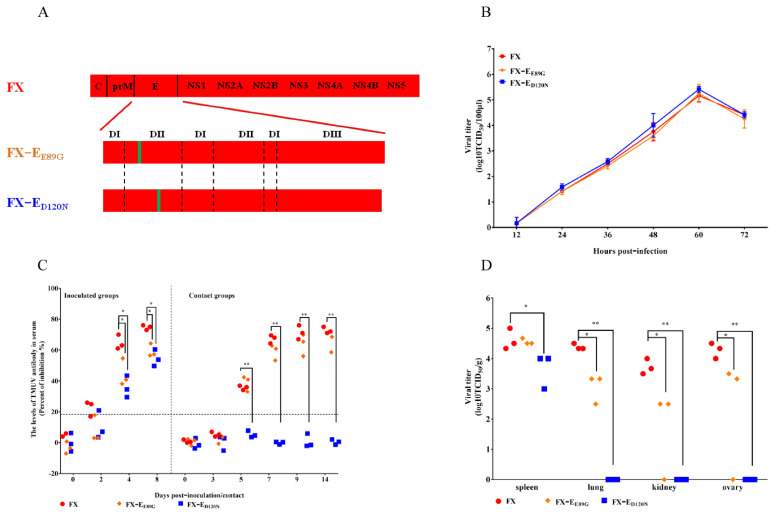
Virus generation, replication, tissue tropism, and transmissibility of parental and mutant TMUVs in vitro and in ducks. (**A**) Schematic diagram of chimeric viruses. The colored bars indicate the origin of the viral protein: red, FX; green, 180P. The viruses were rescued in the background of FX. (**B**) Replication of parental and single amino acid mutant TMUVs. DEF cells were infected by the parental and single amino acid mutant TMUVs at a MOI of 0.001. Virus samples from the supernatant were collected at different time points and titrated on BHK-21 cells. The data for virus titers indicate the means of the results of triplicates. (**C**) Antibodies against TMUV in FX-, FX-E_E89G_-, or FX-E_D120N_-inoculated and contact ducks. Serum was considered positive when the PI value was ≥18.4%. (**D**) Virus titers in different samples of ducks inoculated with FX, FX-E_E89G_, or FX-E_D120N_. The data for virus titers indicate the results of 3 ducks. (* *p* < 0.05; ** *p* < 0.01).

There were only two amino acids mutations in the E-DII domain between FX (E_E89, D120_) and 180P (E_G89, N120_). To determine which amino acid contributed to the change in tissue tropism and transmissibility loss of 180P in ducks, two single amino acid mutant FX-E_E89G_ and FX-E_D120N_ were generated in the background of FX (Figure 3A) and were further characterized in vitro and in ducks. FX-E_E89G_ and FX-E_D120N_ had a similar replication capability as FX on DEF cells infected with each virus at a MOI of 0.001 (Figure 3B). All inoculated ducks in the parental FX and mutant groups showed seroconversion on 4 dpi (Figure 3C). Transmission study results show that FX and FX-E_E89G_ were transmitted to contact ducks; however, the sera of contact ducks in the FX-E_D120N_ group were still negative at 14 dpc (Figure 3C), which indicated that FX-E_D120N_ did not transmit among ducks. Similar virus loads were detected in spleens of ducks inoculated with either FX-E_E89G_ or FX, while lower virus titers were observed in lungs, kidneys, and ovaries of ducks infected by FX-E_E89G_ than by FX (Figure 3D). Moreover, significantly lower viral titers were found in spleens of ducks inoculated with FX-E_D120N_, and no virus was detected in lungs, kidneys, and ovaries of ducks in the FX-E_D120N_ group (Figure 3D). These results indicate that the E_D120N_ mutation restricted the tissue tropism of FX in ducks. The results demonstrate that the E_D120N_ mutation was critical for the tissue tropism and that it abolished transmissibility of TMUV in ducks. 

### 3.4. E_D120N_ Mutation Introduced an Extra High-Mannose Type N-Linked Glycosylation in E Protein of 180P

**Figure 4:**
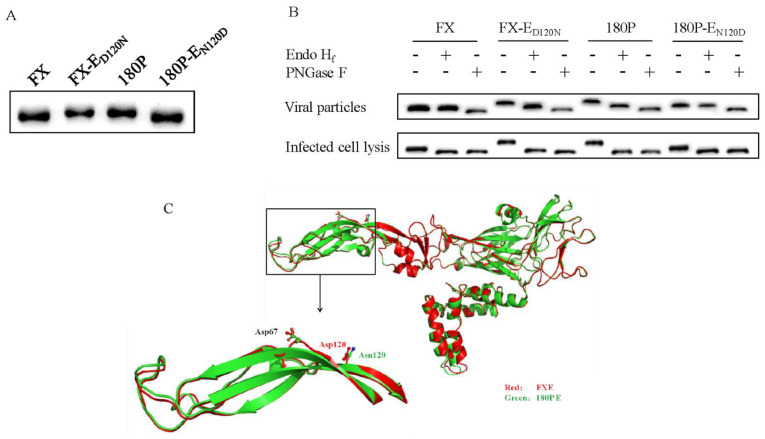
Glycosylation pattern identification. (**A**) Glycosylation status of FX and 180P E protein mutants. The apparent molecular weight of E protein mutants was analyzed using Western blotting against frozen viral stocks. (**B**) N-linked glycosylation patterns identification at N120 and N154. BHK-21 cells were infected with either FX, FX-E_D120N_, 180P, or 180P-E_N120D_. Viruses in supernatants (containing extracellular E proteins) and cell lysates (containing intracellular E proteins) were collected and prepared separately. The samples were digested by either Endo Hf or PNGase F and then were analyzed by Western blotting. Western blots were probed with a monoclonal antibody (2C7) that recognizes TMUV E proteins. (**C**) Constructed homology models of E proteins of TMUVs. To localize glycosylation at position 120 of the E protein, the images of the E proteins of FX and 180P were created with the program Phyre2 using the JEV E protein structure (PDB ID: 5WSN). Homology models of E protein of FX (red) and 180P (green) are shown.

Based on previous studies, there was only one NLG located at position 154 in the TMUV E protein. Meanwhile, the 180P obtained an extra potential NLG site at E120 (120–122 NCT) after obtaining the D120N mutation, which was predicted based on the consensus sequence motif (N-X-S/T) of NLG [20,21]. To confirm whether this N120 was glycosylated, the potential NLG site was knocked out by introducing an N120D mutation in the E protein of 180P, resulting in a mutant virus 180P-E_N120D_ that only containing a single NLG site at N154. On the other hand, FX-E_D120N_ contained two potential NLG sites at N120 and N154. The molecular weights of E proteins of the parental and mutant viruses were analyzed by Western blotting. The results show that the E proteins of FX and 180P-E_N120D_, containing one potential NLG site at N154, had smaller molecular weight than those of FX-E_D120N_ and 180P containing two potential NLG sites at N120 and N154 (Figure 4A), which suggest that the D120N mutation resulted in the introduction of a new glycosylation site at 120 in the E protein of 180P.

To further confirm the N-linked glycosylation pattern at N120 and N154, BHK-21 cells were infected with either FX, FX-E_D120N_, 180P, or 180P-E_N120D_. Viruses in supernatants (containing extracellular E proteins) and cell lysates (containing intracellular E proteins) were collected and treated separately. The samples were digested by either endoglycosidase H_f_ (Endo H_f_, cleaves only high-mannose structures and some specific hybrid structures) or peptide N-glycosidase F (PNGase F, hydrolyzes nearly all types of N-glycan chains from glycopeptides/proteins) and then were analyzed by Western blotting. The intracellular E proteins of the four parental and mutant viruses in cell lysates migrated similarly after being treated with Endo H_f_ or PNGase F, which suggests that the intracellular E proteins were attached with high-mannose glycans at both N120 and N154 sites (Figure 4B). In contrast, the E proteins of FX) and 180P-E_N120D_ in the supernatants were Endo H_f_-resistant but not PNGase F-resistant (Figure 4B), which suggests that the N154 was modified with complex-type glycans. The E proteins of FX-E_D120N_ and 180P, containing two NLG sites at N120 and N154, in the supernatants were partially digested by Endo H_f_ treatment, which indicates that the N120 was a high-mannose type glycosylation. Thus, N-glycosylation of N120 and N154 displayed different types in structure and sugar composition, where high-mannose and complex glycans existed in the E proteins of FX-E_D120N_ and 180P.

## 4. Discussion

Since 2010, TMUV has caused significant economic loss to the duck industry in China [3,4,6,8]. A live attenuated duck TMUV 180P strain was successfully developed as a commercial vaccine by serially passaging a virulent FX strain in CEF [9]. The vaccine 180P strain stimulated humoral antibody at six days post vaccination and provided complete protection against virulent strains. Comparing with wild-type FX, the vaccine 180P was attenuated significantly in viral replication, tissue tropism, and transmissibility in ducks [9], which makes it safe to be used in the field. However, the molecular mechanisms of the attenuation of the 180P vaccine remain unclear. 

To understand genetic determinants for the attenuation of 180P, a series of chimeric and site-mutant viruses were rescued by using reverse genetic technology, and then their virulence and transmissibility were evaluated in ducks. The FX/180PE chimeric virus replicated to significantly lower titers than the wild-type FX in kidney and ovary; in contrast, the replication efficiency of the FX/180PE was significantly higher than that of 180P (Figure 1D), which suggested both E and NS proteins contributed to the replication of duck TMUV. Moreover, the FX/180PE completely abolished the transmissibility compared with the wild-type FX (Figure 1C), which indicates that the E protein is critical for the attenuation and lost transmissibility of the TMUV. Substitution of wild-type FX E with 180P E completely abolished the transmissibility of FX. Further study showed that the D120N mutation located in DII of E contributed to the attenuation and transmissibility of 180P. Introduction of D120N in the E protein significantly attenuated the wild-type FX in ducks. The FX-E_D120N_ replicated only in the spleens of ducks and completely lost the direct-contact transmissibility among ducks (Figure 3). Further analysis of E protein of 180P showed that D120N mutation introduced a potential NLG at 120–122 (NCT) motif, where most of the wild-type TMUV strains contained the “DCT” motif at 120–122 region of E protein. Both Western blotting and PNGase F digestion assay indicated that the E protein of 180P contains two NLGs at the 120–122 and 154–156 positions, while the wild-type FX only contains one NLG at the 154–156 position.

Glycosylation is one of the post-translational modifications and is important to viral glycoprotein functions such as folding, proteolytic processing, and protein trafficking [22]. It is well known that a highly conserved N153 or N154 glycosylation site (depending on the particular virus) is located in E-DI of the flaviviruses [20,21,23,24,25,26], although some flaviviruses lack this NLG site [25,27,28,29,30,31,32]. The NLG at N153 or N154 has been reported to be critical for virulence of many flaviviruses, such as DENV, WNV, JEV, and ZIKV [20,22,24,25,31,32,33,34,35,36,37,38,39,40]. In our previous study, the S156P mutation was reported to abolish the N-linked glycosylation at N154 and to attenuate TMUV among ducks [14], and similar results were observed in DENV2 [41]. Addition of N120 glycosylation (E-DII) or deletion of N154 (E-DI) glycosylation both resulted in transmissibility loss and attenuation of TMUV in ducks, which suggests that N-glycosylation in different domains of E protein have a completely different impact on the virulence of flaviviruses. Similar results were observed in other flaviviruses, where an extra N67 glycosylation attenuated the JEV or WNV [42,43].

There are two types of protein glycosylation distinguished by their attached residues: N-linked is the glycan covalently attached to the asparagine (N) (consensus motif: N-X-S/T except where X is a proline) and O-linked is the glycan attached to the oxygen of serine (S) or threonine (T) residues of the protein. There are three classes of N-linked structures. High-mannose structures contain the unsubstituted terminal mannose sugars that attached to the chitobiose core and typically not to other carbohydrate residues. In complex structures, both the branching and extension of the glycans from the core are typically modified by lactosamine, which can be further modified. Hybrid structures are those in which the core is extended by both the high-mannose arm and one or more lactosamine units [44,45,46]. 

By Western blotting analysis, the results show that N120 and N154 were glycosylated with similar high-mannose type on intracellular E protein. However, N120 and N154 on E protein were glycosylated with different glycan types in extracellular mature viral particles, where N120 was predominantly glycosylated with high-mannose, while glycans at N154 were mainly complex structures. Different from most flaviviruses, DENV E protein contains two N-linked glycosylation sites at N67 located in DII and N153 in DI. The N67 glycosylation site is unique to DENV and has been proposed to interact directly with DC-SIGN, one of the host cell receptors highly expressed in macrophages and dendritic cells [47]. N-glycan structures analysis showed that N67 of E protein was mainly attached by high-mannose, while complex glycans were detected at N153 for DENV. N153 glycans may display a different function from N67 glycans in DENV pathogenesis because only high-mannose on N67 interacted with DC-SIGN, which mediates the viral infection of dendritic cells bearing DC-SIGN receptors and is essential for viral assembly and exit. Loss of the N67 NLG attenuated the DENV [41,47]. 

In the present study, similar to N67 in DENV E, we found N120 at TMUV E was also located in E-DII and that the attached glycans were high-mannose type. However, the addition of the NLG at N120 led to the attenuation and reduced transmissibility of the virus in ducks, which was different from DENV. In contrast, in WNV and JEV, an artificial N-linked glycosylation at N67 of E protein increased the DC-SIGN binding ability and attenuated the virulence of N67 mutants [42,43]. The increased binding ability to DC-SIGN results in high efficiency of presentation to immune cells [48]. Based on structure of E protein referred by homology modeling, the NLG at position 120 was presented and exposed on the surface of DII (Figure 4C), which was close to the NLG at position 67 of E protein of DENV. Whether glycans at N120 of TMUV also interact with duck DC-SIGN protein and attenuate TMUV viruses through a different way, as well as the underlying attenuation mechanisms, needs further investigation.

Taken together, our findings demonstrate that a single D to N mutation at position 120 results in an additional NLG modification and that it was a key determinant of attenuation of the 180P vaccine strain, which elucidates the molecular basis of TMUV attenuation in ducks and provides new insight into the role of NLG in TMUV tissue tropism and transmissibility. The data presented in this study also provide a novel approach for attenuated live flaviviruses vaccine development.

## Figures and Tables

**Table 1:** A total of 19 amino acid mutations scattered throughout the entire viral polyprotein were identified between FX and 180P.

Viruses	Amino Acid Residues																		
	M	E								NS1			NS3	NS4A		NS4B		NS5	
	106 ^a^	37	89	120	155	157	166	312	349	192	205	262	322	54	110	50	112	273	793
FX	A	D	E	D	Y	A	R	V	M	R	K	V	T	F	V	F	S	R	V
180P	V	N	G	N	F	V	K	A	K	G	R	A	I	L	A	Y	L	G	A

^a^ Positions in each viral proteins.
